# Providing monetary and non-monetary goods to research participants: perspectives and practices of researchers and Research Ethics Committees in Zambia

**DOI:** 10.1080/11287462.2018.1527672

**Published:** 2018-10-04

**Authors:** Chris Mweemba, Joseph Ali, Adnan A. Hyder

**Affiliations:** a Department of Health Policy and Management, School of Public Health, University of Zambia, Lusaka, Zambia; b Department of International Health, Johns Hopkins Bloomberg School of Public Health, Baltimore, MD, USA; c Johns Hopkins Berman Institute of Bioethics, Baltimore, MD, USA

**Keywords:** Research ethics, inducements, compensation, monetary and non-monetary goods, Research Ethics Committees, institutional review boards, Zambia

## Abstract

There are disagreements among ethicists on what comprises an “appropriate” good to offer research participants. Debates often focus on the type, quantity, timing, and ethical appropriateness of such offers, particularly in settings where participants may be socio-economically vulnerable, such as in parts of Zambia. This was a Cross-sectional online survey of researchers and Research Ethics Committees (RECs) designed to understand practices, attitudes and policies associated with provision of goods to research participants. Of 122 responding researchers, 69 met eligibility criteria. Responses were also received from five of the six Zambian RECs involved in reviewing research proposals. Forty-nine researchers (71.0%) confirmed previous experience offering goods to participants. Of these, 21 (42.9%) offered participants money only, 18 (36.7%) offered non-monetary goods, while the rest offered both monetary and non-monetary goods. Generally, goods were offered and approved by RECs to compensate for time, lost wages and transportation. One REC and 34.8% of researchers reported being subject to an institutional policy on offering goods to participants. While reimbursement is the main reason for offering goods to participants in Zambia, caution is required when deciding on the type and quantity of goods to offer given the potential for community mistrust and manipulation.

## Introduction

The practice of researchers offering monetary and/or non-monetary goods (referred hereafter generally as “goods,” except where relevant to differentiate) to study participants is common (T. B. Phillips, 2011; Sears, 2001). Various scholars have developed models (e.g. market, working wage, reimbursement and fair share) in an attempt to explain decisions regarding offers to study participants (Dickert & Grady, 1999; T. Phillips, 2011; Ripley, 2006). Despite these efforts, there remains uncertainty about what comprises an “appropriate” good to offer study participants (Council for International & Organizations of Medical Sciences (CIOMS), 2016; T. B. Phillips, 2011), especially in different settings and for different study types. The type, quantity, timing, and ethical appropriateness of various offers are continuously debated (T. B. Phillips, 2011; Ripley, 2006).

Some scholars feel that offering study participants goods, particularly monetary goods, can generate serious ethical concerns (Emanuel, Currie, Herman, & Project Phidisa, 2005; Sears, 2001; Wong & Bernstein, 2011). Beckford and Broome argue that excessive payments to participants may cause individuals to participate in studies without fully considering the risks of the study (Beckford & Broome, 2007). They also maintain that offering payments can cause participants to “withhold information about themselves so as to meet the inclusion criteria for the study” (p.83). It has also been argued that excessive offers can sometimes result in selection bias, as study participants may self-select into studies even when they fail to meet the eligibility criteria (Beckford & Broome, 2007; Ripley, 2006), thereby compromising the scientific validity of the study (Ellenberg, 1994; Grimes & Schulz, 2002) and effectively making it unethical (Emanuel, 2005; Emanuel et al., 2005; Emanuel, Wendler, Killen, & Grady, 2004).

However, others object to the notion that offering goods to participants may compromise the voluntary nature of participation and unduly influence participants (Dickert, Emanuel, & Grady, 2002; Emanuel, 2004, 2005; Emanuel et al., 2005; Wilkinson & Moore, 1997). For example, Wilkinson and Moore (1997) are unconvinced by most arguments opposing payments to study participants, while Emmanuel and colleagues find that undue inducement cannot exist in a research study that has gone through ethical review in which all the study procedures and risks are otherwise deemed acceptable and approved (Emanuel et al., 2005). While there is limited literature distinguishing monetary and non-monetary goods, empirically, some studies that have attempted to show that different types of goods can be viewed differently by research stakeholders. For instance, findings from a study by Molyneux, Mulupi, Mbaabu, and Marsh (2012) revealed that non-monetary goods, such as medical benefits, were preferred because they can minimize the commercialization of research. Apart from medical benefits, such as free screening and treatment, other non-monetary goods offered to participants include food, books, pens clothing etc. (Molyneux et al., 2012).

Debates regarding goods offered to participants persist, with little international guidance (Dickert & Grady, 1999; T. B. Phillips, 2011; Ripley, 2006; Wong & Bernstein, 2011). For instance, even though some international ethical guidelines [e.g. Declaration of Helsinki (World Medical Association, 2008), Belmont Report (Department of Health, Education, and Welfare & National Commission for the Protection of Human Subjects of Biomedical and Behavioral Research, 2014), and the Council for International Organization of Medical Sciences (CIOMS) Guidelines (Council for International & Organizations of Medical Sciences (CIOMS), 2016)] urge ethics review boards to disapprove of research that may be offering inappropriate goods, they do not offer a comprehensive guide on what comprises an “appropriate good”. This lack of guidance can result in variability in both the types and amounts of goods offered to participants (Kimberly, Hoehn, Feudtner, Nelson, & Schreiner, 2006; Latterman & Merz, 2001), which can raise concerns associated with fairness and even exploitation (T. Phillips, 2011).

It is worth noting that most evidence regarding the nature of goods offered to participants has emerged from research in developed countries, and it remains unclear what motivates these researchers in making decisions about offering goods to participants (T. B. Phillips, 2011; Ripley, 2006). Far less has been documented, empirically, about practices, attitudes and policies related to provision of goods to research participants in low- and middle-income countries (LMICs), though concerns have been expressed (Boutin-Foster et al., 2013; Dickert & Grady, 1999; Hyder, Rattani, Krubiner, Bachan, & Tran, 2014; Molyneux et al., 2012; Njue et al., 2015; Njue, Kombe, Mwalukore, Molyneux, & Marsh, 2014). Few studies in sub-Saharan Africa have assessed the types of benefits offered to participants and fairness of such benefits (Molyneux et al., 2012; Njue et al., 2014). These studies focused on benefits offered in studies conducted by the Kenya Medical Research Institute (KEMRI)-Wellcome Trust program in Kilifi, Kenya.

This study is in response to the lack of empirical evidence to guide scholarly debate in this area. We sought to develop an understanding of the factors researchers and research ethics committees (RECs) in Zambia, an LMIC (The World Bank, 2017), consider when making decisions about whether or not to offer goods to study participants. We also examined the type and quantity of goods offered. Approximately 74% of Zambians live on less than 1.25 USD a day (The World Bank, 2015), leaving many research participants potentially economically vulnerable.

## Methods

This study employed a quantitative, descriptive cross-sectional design to investigate researchers’ and RECs’ considerations and practices when making decisions about offering goods to research study participants. Potentially eligible researchers were identified (non-probability, consecutive sampling) from the records of Zambian RECs. To facilitate this, all Zambian RECs known to the study team were given information about the study and permission was sought and obtained to sample researchers through REC records. The RECs provided a list of researchers and email addresses, from their registers, that met the broad eligibility criteria of being a researcher in Zambia and conducting health research involving human participants for at least a year. To capture the perspectives and practices of RECs, all chairpersons of RECs that continuously reviewed research proposals in Zambia during the five years preceding this study were invited to participate. Potential respondents (researchers and REC chairpersons) were invited to complete an online survey (commercial version of SurveyMonkey^©^). Follow-up emails were sent to non-responders every two weeks during the five-month data collection period (02/2016 to 06/2016). Participants who preferred to be interviewed in person were visited and interviewed using the same survey.

### Survey instruments

Two related survey instruments – one for researchers and one for RECs – were developed by faculty of the Johns Hopkins-Fogarty African Bioethics Training Program and the University of Zambia, School of Public Health. The survey instrument for researchers included 36 closed-ended items divided into three parts encompassing: (1) demographics (including filter questions assessing eligibility, e.g. living in Zambia and conducting health research for at least one year), (2) offers made to participants in most recent study, and, (3) challenges and institutional policies around offering goods to participants. The survey for RECs included 16 closed-ended items covering the existence and nature of REC policies on offering goods, and whether the REC had ever requested researchers to offer, revise or remove goods, and reasons behind such requests.

The researcher instrument was pretested with five faculty members of the University of Zambia School of Public Health, while the REC survey was pretested with two members (who were not Chairs) of a REC in Zambia. The survey instruments are available on request.

### Data analysis

Once collected, data were downloaded from SurveyMonkey to Microsoft Excel, where it was checked for completeness and consistency before being exported to STATA 12 (StataCorp, 2011) for analysis. Univariate analysis was conducted to describe the characteristics of researchers and the nature of their experiences with offering goods to study participants, including the type and quantity of goods. Findings were presented using frequencies and percentages. Bivariate analysis was conducted using Fischer's exact test to examine if there were associations between variables of interest.

## Results

### Respondents

#### Researchers

A total of 467 survey invitations were sent to researchers via email. Of the 122 researchers who initiated a survey response, 69 both met the eligibility criteria (i.e. having had lived and conducted health research involving human participants in Zambia for at least one year) and provided complete responses. The response rate for the study was 26.1%; calculated using the American Association for Public Opinion Research (AAPOR) Response Rate Calculator; Response Rate 2 for web-based surveys (AAPOR, 2016).^1^ Forty-six respondents (66.7%) were male; 22 respondents (31.9%) were between 35–44 years old, 22 (31.9%) were between 45–54 years, and 13 (18.8%) and 9 (13%) were aged between 55–64 and 25–34 years, respectively. Nearly two-thirds of respondents (65.2%) were working within public academic institutions, while others were employed in various sectors, including: international non-governmental organizations (NGOs), Zambian NGOs, private academic institutions, and the Zambian government. Table 1 summarizes all respondent demographic characteristics.

**Table 1.** Demographic characteristics of the interviewed investigators.

	*N* = 69	%
Gender		
Male	46	66.7
Female	23	33.3
Age		
25–34	9	13.0
35–44	22	31.9
45–54	22	31.9
55–64	13	18.8
65–74	2	2.9
>75	1	1.5
Employment Sector		
Public Academic Institution	45	65.2
Zambian Non-Governmental Organization	8	11.6
International Non-Government Organization	6	8.7
Private Academic Institution	4	5.8
Other	4	5.8
Zambian Government (Non-Academic/Non-Hospital)	2	2.9

#### Research ethics committees

At the time of this study, there were six RECs involved in reviewing research proposals in Zambia: ERES Converge, Macha Research Trust IRB, Tropical Diseases Research Center Research Ethics Committee, University of Zambia Biomedical Research Ethics Committee, University of Zambia School of Humanities Research Ethics Committee and University of Zambia School of Medicine Research Ethics Committee. All six were invited to participate in the study; five completed the REC survey.

#### Monetary and non-monetary goods offered to participants in Zambia

Of the 69 researchers who completed the survey, 49 (71.0%) had offered research participants monetary and/or non-monetary goods at some point during the five years preceding this study. Out of these 49 researchers, 21 (42.9%) offered participants money only, 18 (36.7%) offered non-monetary goods only, while the rest (*n* = 10; 20.4%) offered both monetary and non-monetary goods. Out of the 20 researchers who did not offer anything to participants, six considered providing goods to participants but could not do so because of multiple reasons which included: lack of resources (*n* = 4), general concern about “undue inducement” (*n* = 3), fear of compromising participants’ ability to think about risks and benefits of the study (*n* = 2), and potential for exploitation of economically vulnerable groups (*n* = 2).

Analyses showed an association between being a male researcher and offering goods to participants (Fischer's exact test P-value = 0.011). About half of the researchers who used a cross-sectional study design had offered goods to participants (24 of the 49 researchers; [49.0%]); however, there was no significant association between the study design and offering goods. Similarly, there was no association between offering goods and researchers’ employment sector. Similarly, the type of good offered was not associated with employment sector, sex, or study design.

Of the 31 researchers who offered money to research participants, 27 (87%) offered approximately $5 (About 50 Zambian Kwacha using the 2016 exchange rate of $1 = K10), 11 offered less than $2 while four offered between $5 and $10. A majority (*n* = 28; 90.3%) cited compensation for participants’ time, transportation and parking as reasons for offering money. Notably, nine researchers (29%) offered money because they were required by an REC to do so. One cited difficulty recruiting as a reason for offering money, and another reasoned that research participants deserved the money as a “minimum wage.” Table 2 summarizes the reasons provided for offering goods to participants.

**Table 2.** Factors considered when offering monetary and non-monetary goods and services to participants.

Money (*N* = 31)			Non-monetary goods (*N* = 28)		
	*N*	%		*N*	%
Compensation for time, transport, parking etc.	28	90.32	Preferred by study participants	16	57.14
Required by a Research Ethics Committee	9	29.03	Advised by the community	10	35.71
Difficulty recruiting	1	3.23	Used previously by self or a colleague	9	32.14
Wage rate	1	3.23	Required by a Research Ethics Committee	5	17.86
** **	** **	** **	Reward participants for their time	2	7.14
** **	** **	** **	Food offered as lunch	2	7.14
** **	** **	** **	Non-monetary good deemed more appropriate	2	7.14
** **	** **	** **	Difficulty recruiting	2	7.14
** **	** **	** **	Study risks	1	3.57
** **	** **	** **	Decision on good to offer done arbitrary	1	3.57

Non-monetary goods offered to participants included food (*n* = 10; 35.7%), transport (*n* = 7; 25%), educational materials (*n* = 6; 21.4%), and clothing (*n* = 3; 10.7%). Other items (*n* = 7; 25%) included soap, washing paste, counseling services, mosquito nets, free malaria screening, deworming tablets for cattle, and newborn baby packs. Slightly more than half of investigators who offered non-monetary participants did so because their participants preferred goods over money, while others (35.7%) indicated that offering non-monetary goods was advised by community representatives such as village heads and chiefs. Other researchers offered non-monetary goods because previous studies in the area, conducted either by themselves or colleagues, had offered non-monetary goods (*n* = 9; 32.1%), while others were requested by an REC to offer non-monetary goods (*n* = 5; 17.8%). In addition, non-monetary goods were sometimes offered to participants to improve recruitment, because of the perceived risks of the study (the nature of the perceived risks was however, not specified by the respondents). Other motivations for offering non-monetary goods, as summarized in Table 2, included, among other things, rewarding participants for their time, as a form of material support for participants, e.g. offering newborn clothing to pregnant mothers etc.

#### Policies on offering goods to research participants in Zambia

All five participating RECs confirmed that their committees review health research involving human participants. Only one REC reported having a specific policy on offering goods to participants. According to the REC, this policy was developed in 2006 and though not enforced, provides guidelines on type and quantity of goods to be offered to participants. Though requested, we were unable to have access to the policy.

Another REC indicated that, even though it did not have a formal policy on goods to participants, it had some “guidelines” on selecting the appropriate goods, but additional information about the nature of these “guidelines” was not provided. Another REC, also without a written policy, indicated that it required all proposed compensation to be included in the protocol submission. All five RECs indicated that they had previously reviewed proposals with offers of goods to participants.

When researchers were asked about the existence of institutional policies on offering goods to participants, 24 (34.8%) reported the existence of such policies within their institutions, while the remainder either reported that policies did not exist (*n* = 28; 40.6%) or were not sure about their existence (*n* = 17; 24.6%). Of the 24 researchers with institutional policies, 12 indicated that the policy provided guidance on both the type and quantity of goods to offer participants, 5 on the type and 4 on quantity of goods only. The remaining 3 researchers mentioned that their policies provided guidance on “other criteria” than the type and quantity of goods. The “other criteria” were not specified.

#### Factors RECs in Zambia consider when deciding whether to approve goods offered to study participants

REC chairpersons were asked various questions to ascertain factors that their respective RECs consider when deciding whether to approve a study offering goods to participants. When asked about goods offered in higher-risk studies relative to lower-risk studies, three out of five RECs felt that, regardless of study risks, there should be no differentiation in the goods offered, while the other two felt that participants in higher-risk studies should receive more goods. The RECs were also asked to indicate under which circumstances they felt offers to participants could become problematic. All five REC representatives felt that offers may be problematic when they cause participants to join studies that they otherwise would not have joined, and when the goods cause participants to ignore study risks that they otherwise would have considered. Four out of five REC chairpersons felt that excessive offers, and offering goods to economically vulnerable participants could become problematic or cause undue influence.

Further, REC chairpersons were asked a multiple response question on whether, in the last five years, they had asked a researcher to offer, revise, or remove goods provided to participants. One REC indicated having requested a researcher to *offer* a good to participants in order to compensate for their lost wages, transportation costs, and due to the large amount of time spent on the study**.** Three RECs confirmed that they had requested a *revision* to a good offered to participants – two RECs requested *decreasing* the amount of a good being offered because it was perceived to be excessive and could potentially induce participants to join the study; one REC requested an *increase* in the quantity of good offered because it deemed the initial offer too little in comparison to the time participants were spending on the study. Another REC indicated that it requested the researcher to *remove* the good proposed because it was thought that it would have compelled individuals to join the study given their perceived socioeconomic conditions. Table 3 summarizes data on availability of written policies by the RECs, and previous requests to offer, revise, or remove goods.

**Table 3.** REC policies and requests to offer, revise or remove a proposed good.

REC	Written policy on offers?	Asked researcher to offer reward?	Reasons	Asked researcher to revise reward?	Reasons	Asked researcher to remove reward?	Reasons
REC #1	Yes	No		Yes	Reward was inducement	Yes	Economic/social conditions of study population – vulnerable to exploitation
REC #2	No	No		No		No	
REC #3	No	No		Yes	Reward too excessive	No	
REC #4	No	No		Yes	Reward too little	No	
REC #5	No	Yes	To compensate for lost wages due to participation To compensation for transport Study location Large amount of time required for participation	No		No	

The five REC chairpersons were also asked what factors researchers should consider when deciding on goods to offer participants. Factors cited included compensation for time, transportation and parking costs, the wage rate, study risks, study location, study duration, study type, and consideration of goods offered by other studies in the study area.

#### Perceived impact and challenges of offering goods to study participants

The 49 researchers who had offered goods to participants were asked about the impact and challenges associated with these offers. Eighteen researchers (36.7%) felt that offering goods to participants improved relations and built trust between researchers and study communities, 12 (24.5%) indicated that offering goods helped retain participants, while 3 felt that the offers helped with recruitment. The remaining 19 researchers (38.8%) did not indicate any impact of goods offered to participants.

Regarding challenges associated with offering goods to participants, nearly two-thirds of researchers who offered goods (*n* = 30; 61.2%) faced no challenges. The remaining 19 researchers cited various challenges, including having participants in some studies demanding more than what was being offered. Three researchers (6.1%) felt that participants who were recruited from potentially vulnerable populations were withholding information to meet eligibility criteria and obtain goods (See Table 4).

**Table 4.** Benefits and challenges of offering goods to participants.

Benefits (*N* = 49)			Challenges (*N* = 49)		
	*N*	%		*N*	%
Helped build community trust	18	36.74	Participants wanting more than offered	8	16.3
Helped with retaining participants	12	24.49	Mistrust by participants/community	4	8.2
Helped with recruitment	4	8.16	False/withholding information to meet eligibility criteria	3	6.1
No reported benefits	15	30.61	Recruitment of potentially vulnerable	2	4.1
** **	** **	** **	Distributional challenges	2	4.1
** **	** **	** **	No reported challenges	30	61.2

## Discussion

This study provides evidence that offering a moderate amount of goods (both monetary and non-monetary) to study participants is a common practice in Zambia. It also suggests that various motivations exist for offering (or choosing not to offer) goods, though money was most frequently reported to be offered to reimburse for expenses incurred, and non-monetary goods were often offered when they were thought to be desired by participants or communities. RECs in the country are likely to face challenges in making determinations comfortably and consistently due to the general absence of detailed policies on provision of goods despite concerns about many participants’ socioeconomic vulnerability. With research on communicable and non-communicable diseases rapidly increasing in Zambia, the number of studies offering goods to participants is also likely to increase. Ultimately, the mandate of RECs should be to develop and strengthen local policies on goods offered to study participants, which will lessen the current back-and-forth between RECs and researchers, on whether to provide goods, and the quantities of goods to provide. The fact that RECs have to request researchers to provide, modify or remove goods is good evidence of variability in goods offered to participants, a problem widely reported by others (Dickert et al., 2002; T. B. Phillips, 2011; Ripley, 2006).

Regarding the reasons for offering goods to participants, our findings are fairly similar to those from high-income countries in which money was commonly offered as reimbursement (Dickert et al., 2002; Fry et al., 2005; Ripley, Macrina, Markowitz, & Gennings, 2010). Results from this study reveal that the reimbursement model mentioned earlier – i.e. payments only covering the participants’ expenses (Dickert & Grady, 1999) – is the main reason researchers in Zambia offer money to participants. While Ripley and colleagues (Ripley et al., 2010) found the reimbursement model to be the least used model among researchers in the US when deciding on offers to participants, the model was the most favored among IRBs in their study, and in this study as well. While models are important in guiding decisions on offering goods to participants, they offer little guidance on the quantity of good to be offered (T. B. Phillips, 2011; Ripley, 2006). A traditional wage compensation model, i.e. paying participants a standard wage proportionate to that paid for unskilled but essential jobs (Dickert & Grady, 1999), could be a good starting point to compute quantities of goods. However, the feasibility of this approach remains challenging, especially for studies and in settings where minimum wage policies do not exist, such as studies involving prisoners and children.

Further, while related studies in high-income countries have focused more on monetary payments to participants, this study has highlighted the importance of non-monetary offers to participants in Zambia, and possibly in other LMICs. The study revealed that, unlike monetary offers, which are often determined by researchers and REC members, non-monetary offers are mainly advised by participants and their communities. The results suggest that, providing non-monetary offers (i.e. food offers, transportation, and educational materials) could be an important step in enhancing collaborative partnerships between researchers and communities, especially in the context of LMICs countries where monetary offers seem to evoke superstitions (Mwinga & Moodley, 2015; Zulu et al., 2014). Such collaborative partnerships are a necessary prerequisite for ethical research (Emanuel et al., 2004). In view of these findings, a new “community-driven” model (i.e. where decisions about goods being offered are advised by communities), could be added to existing models to help guide decisions on goods offered to participants, especially in the context of LMICs.

Despite non-monetary offers being important in Zambia, and possibly other LMICs, care should be taken to ensure that research participants are adequately compensated, particularly for any expenses incurred, to avoid exploitation. Equally important is ensuring that community consultations are comprehensive and include discussion of what both parties consider to be *fair* offers – monetary and/or non-monetary. This can support efforts to move away from paternalistic processes for determining what is in the best interest of participants or communities involved (Burhansstipano, Christopher, & Schumacher, 2005). Our study revealed an association between male researchers and offering goods to participants; however, these findings are preliminary and future research is required to assess whether such trends exist in other contexts. Additionally, the non-probability design may have not have controlled for inherent biases present in the sample.

This study identified the absence of guiding principles on goods offered to participants in Zambia, and showed similar gaps in international ethical guidelines and regulations on research involving human participants (i.e. the Declaration of Helsinki, CIOMS Guidelines and the Belmont Report) (Council for International & Organizations of Medical Sciences (CIOMS), 2016; Department of Health, Education, and Welfare & National Commission for the Protection of Human Subjects of Biomedical and Behavioral Research, 2014; World Medical Association, 2013). Due, in part, to these inadequacies, RECs in Zambia have previously required researchers to add, revise, or remove a good offered to participants, without giving explicit guidance on the type and quantity to be offered. An opportunity exists for RECs and researchers to collaborate on developing policies that will promote ethical research, as advocated elsewhere (Eissenberg et al., 2006; Emanuel et al., 2004), and this is crucial for settings such as Zambia where some researchers report having no or relatively sparse institutional policies on offering goods to participants.

Offering goods to participants can have both positive and negative impacts in Zambia. For instance, some researchers felt that goods offered to participants could improve relationships and build trust between researchers and study communities. A robust understanding of this can potentially improve collaborations and increase the credibility and uptake of interventions and findings. However, conclusions about the impact of offers to participants may be premature until additional research about participants’ perspectives is undertaken to ascertain the extent to which these sentiments are mutually felt and their limits. This study also highlights that offering research participants goods is thought to improve recruitment in Zambia, a finding consistent with data from Australia (Fry et al., 2005), where some researchers paid participants money due to anticipated recruitment difficulties. Similarly, a study in the USA (Ripley et al., 2010) revealed that, researchers and IRB chairpersons rated recruitment difficulty as an important factor in determining payments to participants. The above findings support the “market model,” that is, offering goods to improve recruitment and retain participants (Ripley et al., 2010). Others, however, may argue that, offering goods to improve recruitment or retain participants could create ethical concerns because participants may join studies that they otherwise would not have joined (Dickert & Grady, 1999; Macklin, 1981; McGee, 1997).

Finally, a feeling among some researchers that offering goods to participants resulted in suspicion and mistrust between researchers and communities in Zambia must also be taken seriously. Misconceptions and suspicions of research seem to be a common occurrence in Zambia, as has been documented by others (Mwinga & Moodley, 2015; Zulu et al., 2014). Potential researchers in the country need to engage with this seemingly conflicting situation in which provision of goods has the potential to enhance collaborative partnerships and also contribute to mistrust. Again, further research is required to assess the impact of goods offered in research. Lastly, we found a concern amongst three researchers (6.1%) in Zambia that, goods offered to participants could potentially cause individuals to withhold critical information about themselves to meet the eligibility criteria, as shown elsewhere(Beckford & Broome, 2007; Ripley, 2006), but our study was not designed to document the extent to which this occurs in practice. Further research on this issue may be needed as it has the potential to be a concern for participant safety and research validity (Department of Health, Education, and Welfare & National Commission for the Protection of Human Subjects of Biomedical and Behavioral Research, 2014; World Medical Association, 2013).

## Limitations

This study may be limited in its generalizability. It employed an online survey to collect data and was limited to researchers whose email addresses were in the records of Zambian RECs which were used as a sampling frame for the study. The study was also limited to researchers who had internet access during the data collection period. The response rate for the study was very low, but this is consistent with other web based surveys elsewhere (Guo, Kopec, Cibere, Li, & Goldsmith, 2016; Hardigan, Succar, & Fleisher, 2012; Reed, Crawford, Couper, Cave, & Haefner, 2004; Sax, Gilmartin, & Bryant, 2003).

## Conclusion

This study is a novel effort to profile the practices of researchers and RECs in Zambia regarding monetary and non-monetary goods offered to study participants. Similar to practices in high-income countries, offering goods to participants is a common practice in Zambia. However, considering that most participants in LMICs are likely to be economically disadvantaged, it is the responsibility of both the RECs and researchers to carefully consider the nature, type and amount of good offered for each study. In addition, fostering collaborative partnerships between RECs, researchers and communities could be important for trust-building, and may enhance the ethical quality of goods provided, and practices more generally, in research.

While reimbursing participants seems to be a key reason for offering goods in Zambia, caution should still be exercised when deciding on the type and quantity of goods to offer given the potential for community mistrust and manipulation. Computing quantities of goods to offer participants remains a challenge for both researchers and RECs, and this study recommends that monetary reimbursements in Zambia be guided by actual expenses and/or the prevailing wage, while non-monetary reimbursements be determined consultatively with communities and community leaders in the study areas. This recommendation does not eliminate the need for RECs, researchers, and study communities to engage in a consultative process to develop policy guidelines on goods offered to participants. It is hoped that future research can build from this initial work to enhance understanding of some of the associations that we have uncovered, and identify contextually appropriate “best practices” in the provision of monetary/non-monetary goods to study participants.

It is important to note that awareness of the nature of practice and of ethical issues associated with offering goods to participants, on their own, are unlikely to materialize into country guidelines for Zambia, or any other country, without additional research and proactive efforts from relevant stakeholders. We hope this study stimulates similar and related work in other LMICs.

## Note

1 AAPOR Response Rate 2 is calculated using the formula; (I + P)/(I + P) + (R + NC + O) + (UH + UO), where I = Complete Interviews, P = Partial Interviews, R = Refusal and break off, NC = Non-Contact, O = Other, UH = Unknown Household, UO = Unknown Other.
